# Serum Tie-1 is a Valuable Marker for Predicting the Progression and Prognosis of Cervical Cancer

**DOI:** 10.3389/pore.2021.1610006

**Published:** 2021-12-17

**Authors:** Rui Bai, Bowen Diao, Kaili Li, Xiaohan Xu, Ping Yang

**Affiliations:** ^1^ Department of Obstetrics and Gynecology, First Affiliated Hospital, School of Medicine, Shihezi University, Shihezi, China; ^2^ The NHC Key Laboratory of Prevention and Treatment of Central Asia High Incidence Diseases, First Affiliated Hospital, School of Medicine, Shihezi University, Shihezi, China; ^3^ Department of Gynecology, Xinrui Hospital of Xinwu District, Wuxi, China

**Keywords:** survival, cervical cancer, diagnostic biomarker, Tie-1, angiopoietin

## Abstract

**Objective:** To investigate whether serum Tie-1 (sTie-1) is a valuable marker for predicting progression and prognosis of cervical cancer.

**Methods:** Enzyme-linked immunosorbent assay (ELISA) was used to detect serum sTie-1 concentrations in 75 cervical cancer patients, 40 cervical intraepithelial neoplasia (CIN) patients, and 55 healthy controls without cervical lesions, and sTie-1 levels were compared between the groups. Receiver operating characteristic curves was used to evaluate the diagnostic value of sTie-1. The relationship between sTie-1 concentrations in patients with cervical cancer and clinicopathological features and prognosis were analyzed, and the risk factors for postoperative recurrence were determined using univariate and multivariable Cox proportional hazards regression.

**Results:** We found that sTie-1 concentrations gradually increased according to lesion severity (i.e., cancer vs. CIN; *p* < 0.05) and were significantly elevated in adenocarcinoma compared with healthy controls. sTie-1 levels strongly distinguished between cervical cancer patients and the healthy controls (area under the curve = 0.846; cut-off value = 1,882.64 pg/ml; sensitivity = 74.6%; specificity = 96.4%). Moreover, sTie-1 levels in cervical cancer patients were significantly associated with tumor size, advanced tumor stage, lymph node metastasis, and reduced 4-years progression-free survival. Cervical cancer patients with high sTie-1 concentrations had a 3.123-fold [95% confidence interval (CI): 1.087–8.971, *p* = 0.034] higher risk for tumor recurrence.

**Conclusions:** Elevated sTie-1 levels in patients with cervical carcinoma were associated with tumor progression and poor prognosis, indicating that sTie-1 may be a valuable marker for predicting progression and prognosis of cervical cancer.

## Introduction

Cervical cancer is the fourth most common cancer in women and also is one of the leading causes of cancer related death in women [1]. Approximately 29,500 women die due to cervical cancer in China each year, accounting for approximately 11% of cervical cancer deaths worldwide [2]. Cervical fluid-based cytology and human papilloma virus (HPV) testing can effectively screen for cervical cancer. However, clinicians are always concerned about whether there may be a simpler and more convenient method, such as serum detection, that can detect cervical cancer early and help monitor tumor progression more effectively.

Recently, the angiopoietin (Ang)/tyrosine kinase receptor (Tie) system, the second largest angiogenic system after vascular endothelial growth factor; (VEGF), has attracted increasing attention because targeting VEGF has demonstrated limited efficacy in cervical cancer detection and monitoring [3]. Ang/Tie signaling mainly includes two ligands (Ang-1 and Ang-2) and two receptors (Tie-1 and Tie-2), which are expressed in endothelial cells (ECs). Ang-1 and Ang-2 both bind directly to Tie-2, thus playing a critical role in vascular development, physiological and pathological angiogenesis, and cancer cell invasion and metastasis [4,5]. Tie-1 and Tie-2 are homologous receptors, their extracellular amino acid sequence consistency is 33%, while that of the intracellular region is 76%. However, the ligand for Tie-1 has not been determined thus far [3,6], and research on its function and related mechanisms has been progressing slowly. Current studies suggest that Tie-1 may be a key factor in regulating the Ang/Tie pathway.

Most studies on Tie-1 have focused on its function and related mechanisms within endothelial angiogenesis. In physiological angiogenesis, Tie-1 can bind directly to Tie-2 to form polymers, reduce the internalization of phosphorylated Tie-2 (pTie-2) and protein degradation (lysosomal pathway), stabilize the phosphorylation signal for Tie-2, and positively regulate Ang/Tie-2 signaling to promote the survival and migration of endothelial cells [7]. In tumor angiogenesis, the deletion of the Tie-1 gene in ECs was found to inhibit the phosphorylation level at Tie-2 y1106 and reduce the migration ability of ECs [8]. Downregulation of Tie-1 expression in ECs can inhibit tumor growth, and Tie-1 depletion can improve the anti-tumor angiogenesis effect of Ang/Tie-2 targeted therapy [9].

However, research on the expression of Tie-1 in tumors and its association with the clinicopathological characteristics and prognosis of tumor patients, as well as the mechanisms mediating its role in tumor progression, have not been well studied. A few studies have demonstrated that Tie-1 is highly expressed in tumor cells in some cases of invasive ductal carcinoma of the breast, and is positively associated with the rate of lymph node metastasis [10]. In colorectal adenocarcinoma cells, Tie-1 expression is positively correlated with depth of invasion, tumor stage, and lymph node metastasis [11]. A recent study reported that high baseline circulating Tie-1 levels in breast cancer predict a worse prognosis [10]. Our previous studies have shown that Tie-1 is highly expressed in cervical squamous cancer cells as compared with normal squamous cells [5]. However, the clinical significance of circulating Tie-1 in cervical cancer remains unclear. Therefore, in this study, we investigated whether serum Tie-1 (sTie-1) is a valuable marker for predicting the progression and prognosis of cervical cancer.

## Materials and Methods

We conducted a single-arm prospective study, with serum samples collected from 75 patients with cervical cancer, 40 cervical intraepithelial neoplasia (CIN) patients, and 55 healthy women (controls) presenting for primary care at the Department of Gynecology at the First Affiliated Hospital of Shihezi University School of Medicine between August 2017 and August 2020. The 75 cases of cervical carcinoma included 63 patients with cervical squamous cell carcinoma (CSCC) and 12 with cervical adenocarcinoma (CADC). The 40 cases of CIN included 17 cases of CIN2 and 23 cases of CIN3. Each diagnosis of cervical cancer was made by two or more clinicians (i.e., oncologists and pathologists) at the hospital. The prognostic value for sTie-1 in cervical cancer patients was evaluated according to the patients’ follow-up data as of October 2021. The median follow-up time was 30 months (range, 6–56 months). Among the 75 patients, 18 had a recurrence and 10 died. Patients with inflammation (i.e., abnormally elevated levels of C-reactive protein or procalcitonin), other types of cancer, connective tissue disease, or atherosclerosis (determined based on anamnestic data or abnormal imaging studies) were excluded from the current study. The average age and age range of patients of normal group were 53 (range, 28–72) years, the average age and range of patients in the CIN group were 51 (range, 41–69) years, and the average age and range of cervical cancer group were 55 (range, 35–83) years. There was no significant difference in the age of the three groups by one way ANOVA (F = 2.607, *p* = 0.077). This study was approved by the hospital ethics committee at our medical center (approval number: 201704001) and written informed consent was obtained from all enrolled participants. This research was conducted in accordance with the principles of the Declaration of Helsinki.

### The Clinicopathological Characteristics of Cervical Cancer Patients and Clinical Definitions

The clinicopathological information of 75 cervical cancer patients was collected, including the patients’ age at the time of diagnosis, FIGO stage (2018), tumor size, pathological type, differentiation, whether there was lymph node metastasis (LNM), lympho-vascular space invasion (LVSI), and whether the infiltration depth of cervical stromal invasion exceeded 1/2. FIGO stage and tumor size were determined by two chief physicians of gynecological oncology, and the tumor size refers to the maximum diameter of the tumor. Among these patients, 46 patients with early stage (IA1-IIA2) underwent surgical treatment, and data on LNM, cervical muscular layer invasion, and LVSI were collected according to postoperative pathological examination report. For 29 patients with advanced cervical cancer, MRI results were used to determine whether LNM was present or whether cervical muscular layer invasion was greater than 50%. Patients were followed up according to the FIGO guideline. Progression free survival (PFS) refers to the time from the first day of treatment (surgery or radiotherapy or chemotherapy or concurrent chemoradiotherapy) to the date when there is evidence of tumor recurrence or the last follow-up. Overall Survival (OS) means the time that from the first day of treatment to death for any cause or to the date of last contact. The time is calculated by month. Patient characteristics were described in Table 1.

**TABLE 1:** Association between sTie-1 concentration and the clinicopathological characteristics of cervical cancer patients (n = 75).

Parameters	Case, n (%)	sTie-1 (pg/ml)^a^	*p*
Age, year			
<50	27 (36.0)	1918.44 (1875.11–2063.82)	0.551
≥50	48 (64.0)	1935.38 (1885.00–2129.97)	
FIGO stage (2018)			
1A1–IIA2	46 (61.3)	1913.62 (1492.36–2046.95)	**0.021**
≥IIB	29 (38.7)	1942.69 (1904.90–2197.05)	
Tumor size			
<2 cm	21 (38.9)	1880.27 (1468.85–1940.78)	**0.006**
≥2 cm	54 (61.1)	1952.51 (1897.71–2122.13)	
Pathological type			
Squamous carcinoma	63 (84.0)	1924.17 (1880.27–2086.57)	0.885
Adenocarcinoma	12 (16.0)	1913.78 (1877.91–2123.49)	
Differentiation			
Poor	19 (25.6)	1917.31 (1885.01–2086.57)	0.908
Well and moderate	56 (74.4)	1928.56 (1875.22–2114.97)	
Lymph node metastasis			
Negative	52 (69.3)	1913.62 (1821.48–2044.45)	**0.037**
Positive	23 (30.7)	2002.14 (1908.82–2163.03)	
Cervical stromal invasion			
<1/2	42 (56.0)	1911.68 (1869.06–2069.51)	0.162
≥1/2	33 (44.0)	1968.41 (1890.32–2143.60)	
Lymph vascular space invasion^b^			
Negative	35 (76.1)	1887.28 (1486.35–2041.33)	0.279
Positive	11 (23.9)	1984.00 (1812.11–2150.41)	

a Data are expressed as the median (P25–P75).

b Data for 46 patients.

Bold font indicates significant difference.

### Serum Samples and ELISA Assay of sTie-1

Serum samples from patients with CIN and cervical cancer were collected at first admission prior to any treatment. Briefly, fasting venous blood (3–4 ml) was collected in the morning for all participants. The samples were centrifuged at 3,000 G/min for 10 min. The sera were collected and the supernatants were extracted, placed in a 1.5 ml Eppendorf tube, and stored in a freezer at −80°C for future use. A standardized sandwich enzyme-linked immunosorbent assay (ELISA) was used to detect sTie-1 concentrations with a human serum Tie-1 test kit purchased from RayBiotech (Peachtree Corners, GA, United States). The assay was performed in strict accordance with manufacturer instructions; and each serum specimen was assayed twice. If the sTie1 values were discrepant by 10% or more, the samples were retested.

### Statistical Analysis

Statistical Package for the Social Sciences (SPSS) software (version 22.0; SPSS, Inc., Chicago, IL, United States) was used for all statistical analyses. Continuous variables not conforming to a normal distribution were described as medians and interquartile ranges. The differences in serum Tie-1 levels between the three aforementioned groups were analyzed using nonparametric tests, including the Kruskal–Wallis (K-W) test, the Jonckheere-Terpstra (J-T) test, and the Mann–Whitney (M-W) U test. Chi-square tests were used to compare rates between patients with cervical cancer and CIN. Receiver operating characteristic (ROC) curve analysis was performed to analyze the diagnostic value of sTie-1. Kaplan–Meier survival curves were used to predict patient survival. Cox univariate and multivariate proportional hazard models were used to determine the risk factors affecting the prognosis of patients with cervical cancer. All tests were two-tailed, and *p* values < 0.05 were considered statistically significant.

## Results

### Cervical Lesion Severity and sTie-1 Concentrations

**FIGURE 1:**
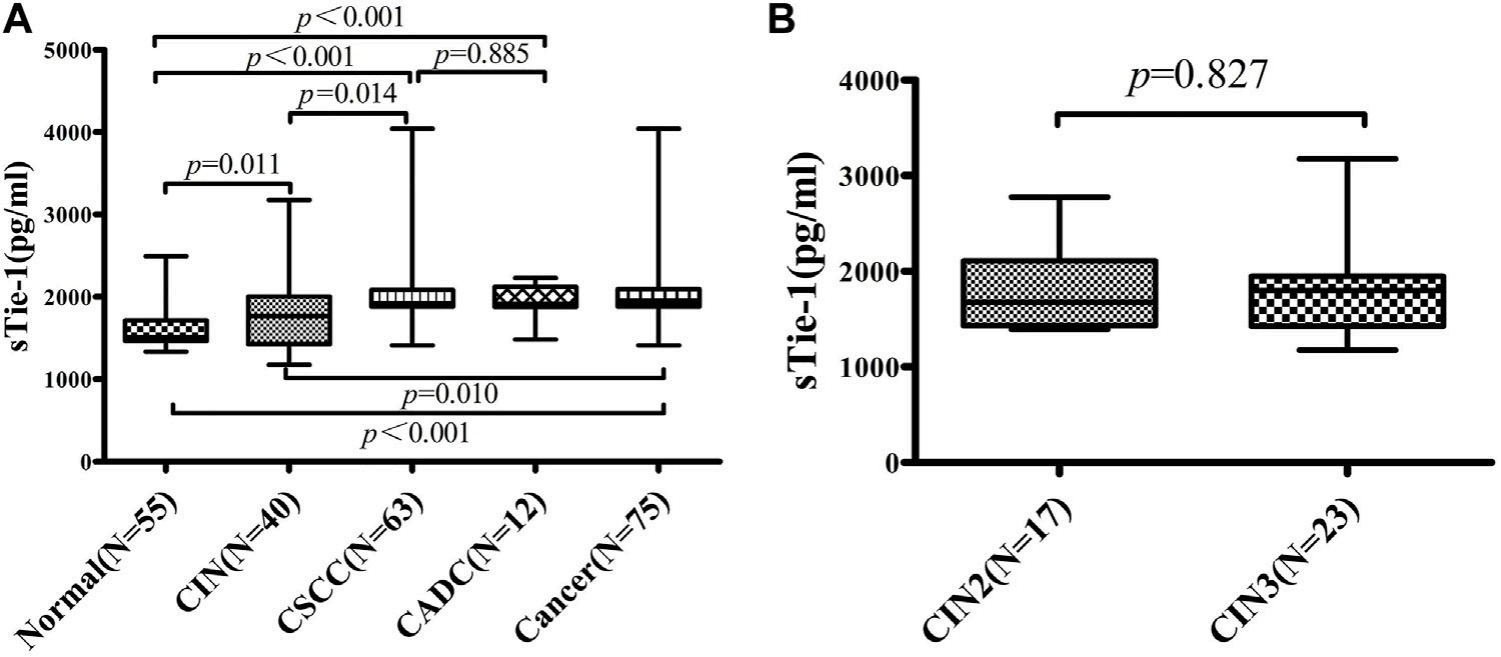
sTie-1 concentrations are altered in patients with cervical neoplasia. sTie-1 concentration in 55 patients of normal control, 40 CIN patients, and 75 cervical cancer patients were determined by ELISA. The differences of sTie-1 between groups were shown **(A)**, and the differences of sTie-1 between CIN2 and CIN3 were shown **(B)**. CSCC, cervical squamous cell carcinoma; CADC, cervical adenocarcinoma; CIN, cervical intraepithelial neoplasia.

The sTie-1 concentrations of 55 healthy controls, 40 CIN patients, and 75 cervical cancer patients were detected by ELISA. For controls, CIN patients, and cervical cancer patients, the median concentrations of sTie-1 were 1,510.15 pg/ml (interquartile range: 1,466.85–1,713.05), 1,806.20 pg/ml (1,429.02–2,003.15), and 1,924.17 pg/ml (1,880.27–2,095.53), respectively. The K-W test showed that, sTie-1 levels gradually increased with an increase in the severity of cervical lesions (*p* < 0.05) (Figure 1A). Moreover, sTie-1 concentrations were statistically significantly higher in cervical cancer patients (1,924.17 pg/ml, 1,880.27–2,095.53) and CSCC patients (1,924.17 pg/ml, 1,880.27–2,086.57) as compared with healthy women (1,510.15 pg/ml, 1,466.85, 1,713.05; *p* < 0.001) (Figure 1A), and in CIN patients as compared with the healthy women (*p* = 0.011) (Figure 1A), and in cervical cancer patients and CSCC patients as compared with the CIN patients (*p* = 0.010, *p* = 0.014) (Figure 1A). However, there were no statistically significant differences detected between patients with CADC and CSCC (*p* = 0.885) (Figure 1A), and there were also no statistically significant differences between patients with CIN2 and CIN3 (*p* = 0.827) in the current study (Figure 1B).

### sTie-1 Concentrations and ROC Curves

**FIGURE 2:**
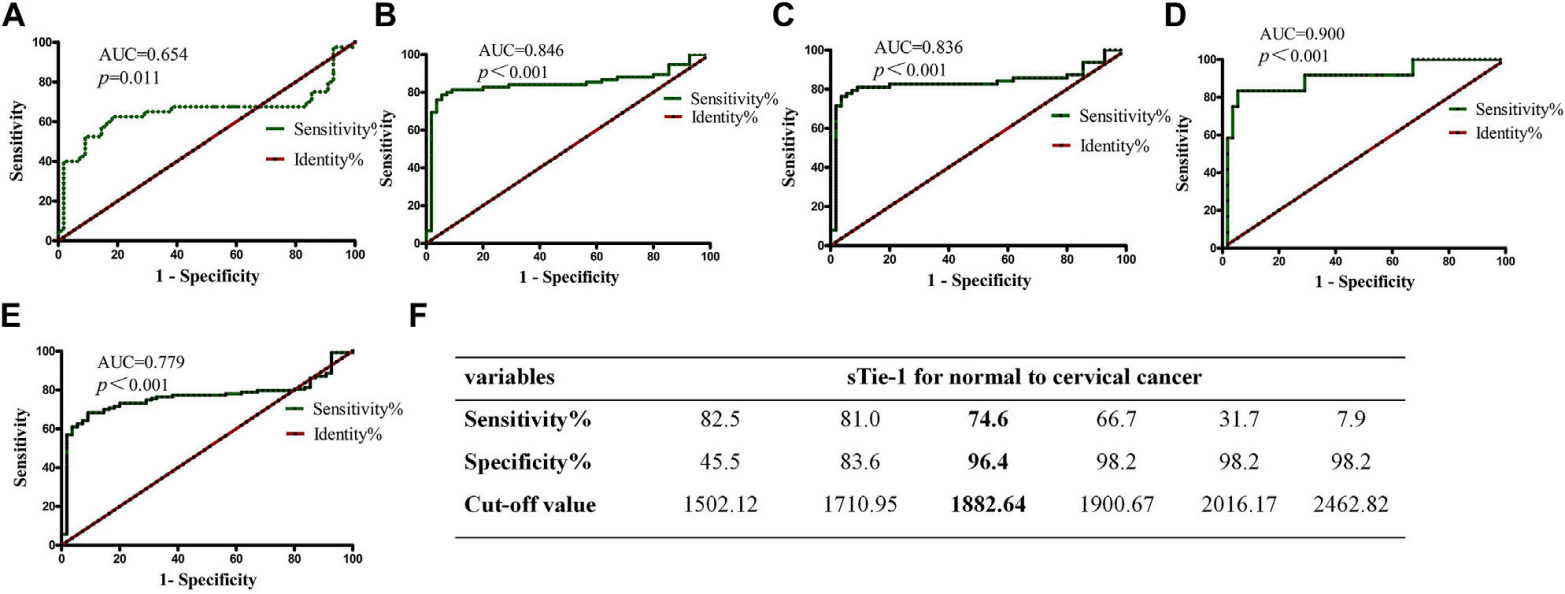
sTie-1 ratio is valuable diagnostic biomarkers for cervical lesions. The ROC curves of sTie-1 for discriminating CIN from normal **(A)**, cervical cancer from normal **(B)**, CSCC from normal **(C)**, CADC from normal **(D)**, cervical cancer and CIN from normal **(E)**. The cut-off values of sTie-1 and corresponding sensitivity and specificity to distinguish cervical cancer from normal were shown **(F)**.

ROC curve analysis was performed to analyze whether sTie-1 could be used as a biomarker to effectively distinguish CSCC cases from CIN cases and healthy controls, as well as to distinguish CIN cases from healthy controls. We found that the area under the curve (AUC) values for sTie-1 [distinguishing CIN cases (Figure 2A), cervical cancer cases (Figure 2B), CSCC cases (Figure 2C), CADC cases (Figure 2D), combined CIN and cervical cancer cases (Figure 2E) from healthy controls] were 0.654, 0.846, 0.836, 0.900, and, 0.779 respectively. The AUC distinguishing cervical cancer cases from healthy was largest (0.846) at the sTie-1 cut-off value of 1,882.64 pg/ml, with a sensitivity of 74.6% and a specificity of 96.4% (Figure 2F).

### Associations Between sTie-1 Levels and the Clinicopathological Characteristics

We analyzed the associations between sTie-1 levels in cervical cancer patients and clinicopathological characteristics. We found that sTie-1 concentration was significantly higher in groups of stage ≥IIB, tumor size ≥2 cm and lymph node metastasis positive than that of stage IA1–IIA2, tumor size <2 cm and lymph node metastasis negative respectively, *p* values were all less than 0.05. Elevated Tie-1 concentration was no significant association with age, pathological type, differentiation, cervical stromal invasion and LVSI (Table 1).

### Higher sTie-1 Predicts Poorer Prognosis in Cervical Cancer Patients

**FIGURE 3:**
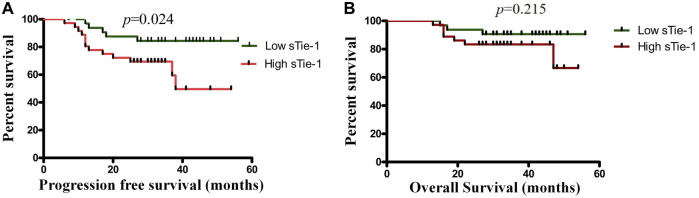
High sTie-1 ratio predicts poorer survival in cervical cancer patients. Kaplan-Meier survival analysis of the progression-free survival **(A)** and the overall survival **(B)** among 75 cervical cancer patients stratified by sTie-1.

Patients were divided into high (n = 38) and low (n = 37) concentration groups according to the median sTie-1 level (1,882.64 pg/ml). In the group of patients with high sTie-1 levels, 13 patients had recurrence (13/38, 34.2%) and 7 died (7/38, 18.4%); 4-years PFS and 4-years OS were 65.8 and 81.6%, respectively. In the low sTie-1 group, 5/37 (13.5%) patients experienced recurrence and 3/37 (8.1%) patients died; 4-years PFS and 4-years OS were 86.5 and 91.9%, respectively. PFS was shorter in the high sTie-1 group as compared with the low sTie-1 group (4-years PFS, 65.8 vs. 86.5%, *p* = 0.024) (Figure 3A). Differences were not statistically significant for OS (*p* = 0.215) (Figure 3B).

### Cox Univariate and Multivariate Analysis

A Cox univariate analysis model was used to analyze the risk factors for 4-years PFS in cervical cancer patients. Statistically significant predictors were FIGO stage ≥IIB (95% CI: 1.832–14.544, *p* = 0.001), poor differentiation (95% CI: 1.270–8.263, *p* = 0.014), and sTie-1 levels (95% CI: 1.093–8.731, *p* = 0.024). We also observed a marginal association between PFS in patients with cervical cancer with a tumor size ≥2 cm (95% CI: 0.856–16.226, *p* = 0.058). These factors were included in the Cox proportional hazards regression model in the multivariate analysis. The most important risk factors for tumor recurrence in cervical cancer patients were a FIGO stage ≥IIB, poor differentiated histopathology, and a high sTie-1 concentration, with adjusted hazard ratios of 5.161 (95% CI: 1.828–14.576, *p* = 0.002), 2.996 (95% CI: 1.154–7.781, *p* = 0.024) and 3.123 (95% CI: 1.087–8.971, *p* = 0.034), respectively (Table 2).

**TABLE 2:** Results of Cox univariate and multivariate analysis of the prognosis of cervical cancer patients (n = 75).

	PFS					
	Univariate			Multivariate		
	HR	*p*	95% CI	HR	*p*	95% CI
Age (year)	1.589	0.380	0.566–4.464	NA		
(<50 vs. ≥50)						
FIGO stage	5.162	**0.001**	1.832–14.544	5.161	**0.002**	1.828–14.576
(≤ IIA2 vs. >IIB)						
Tumor size	3.726	0.058	0.856–16.226	1.129	0.883	0.224–5.685
(<2 cm vs. ≥2 cm)						
Pathological type	1.175	0.565	0.674–2.049	NA		
(CSCC vs. CADC)						
Differentiation	3.239	**0.014**	1.270–8.263	2.996	**0.024**	1.154–7.781
(well and moderate vs. Poor)						
LNM	1.992	0.138	0.783–5.070	NA		
(Negative vs. positive)						
LVSI	2.909	0.230	0.466–18.166	NA		
(Negative vs. positive)						
Cervical stromal invasion	1.450	0.429	0.570–3.688	NA		
(<1/2 vs. ≥1/2)						
sTie-1	3.089	**0.024**	1.093–8.731	3.123	**0.034**	1.087–8.971
(low vs. High)						

LNM, lymph node metastasis; LVSI, lympho-vascular space invasion; HR, hazard ratio; PFS, progression-free survival; NA, not included in multi-factor analysis; 95% CI, 95% confidence interval; HR, risk ratio.

Bold font indicates significant difference.

## Discussion

Cervical cancer is one of the most frequently occurring malignancies in women, particularly metastatic cervical cancer resulting in a poor prognosis [12,13]. In our study, we found that the expression of sTie-1 gradually increased with an increase in the severity of cervical lesions, and that a sTie-1 cut-off value of 1882.64 pg/ml could effectively distinguish cervical cancer patients from healthy controls. Patients with elevated sTie-1 levels showed statistically significant associations with advanced FIGO stage, larger tumor size, and pelvic lymph node metastasis, and higher sTie-1 levels predicted poorer prognosis for patients with cervical cancer. These results suggest that serum Tie-1 may be a valuable biological marker for predicting progression and prognosis in cervical cancer.

The mechanisms of action mediating the effects of Tie-1 in tumor growth and progression are currently a hot topic in the medical research community. Recent research has shown that acute and chronic inflammation leads to cleavage of the Tie-1 ectodomain in ECs, resulting in increased levels of the soluble extracellular domain of Tie-1 and decreased levels of the intracellular domain, transforming Ang-2 from a Ang-1/Tie-2 agonist to an antagonist and initiating a positive feedback loop through FOXO1-driven Ang-2 expression, thereby stimulating pathological angiogenesis [14]. It is well known that the systemic effects of tumors are similar to those of general inflammation. In a study on breast cancer, the median circulating plasma Tie-1 level of metastatic patients was statistically significantly higher than that of healthy controls [10], and our previous experiment showed that cervical cancer cell lines secrete soluble Tie-1 (similar to ECs) [5]. Consistent with these studies, we found that the median level of sTie-1 was statistically significantly increased in patients with cervical cancer as compared with CIN patients and healthy controls in the current study, and that cervical cancer patients with higher sTie-1 levels had poorer survival. A question of interest is that of the role of mediating mechanisms in cervical cancer, which may be similar to those seen in inflammation, with tumor conditions potentially leading to Tie-1 extracellular cleavage and activating the ANG/Tie-2 pathway, resulting in tumor angiogenesis. As mentioned above, previous studies have shown that cervical cancer cells can express Tie-1 at levels similar to ECs, leading to the question of whether Tie-1 cleavage mainly occurs in endothelial cells or in cancer cells. The specific mechanisms mediating these observations are worth exploring in depth in future research.

To the best of our knowledge, Tie-1 studies in human tumors are limited. In breast cancer, enhanced expression of sTie-1 is positively correlated with lymphatic metastasis and shorter survival [10] in hepatocellular carcinoma (HCC) patients, higher Tie-1 expression predicts poorer prognosis [15]. Our study demonstrates that cervical cancer patients with higher sTie-1 levels have larger tumors, more advanced progression, a greater likelihood of lymph node metastasis, and shorter PFS, and a multivariate-adjusted Cox proportional hazards model indicated that cervical cancer patients with high sTie-1 levels were 3.123 times more likely to have a recurrence than those with low sTie-1 levels. Additionally, previous research has found that Tie-1 deletion in experimental murine metastasis models prevents extravasation of tumor cells into the lungs and reduces metastatic foci [9]. These studies indicate that Tie-1 may play an independent role in tumor growth and progression, and may thus be an attractive novel target for cervical cancer therapy. Moreover, in colorectal cancer [11], Tie-1 positive cells were enriched in tumor cells with cancer stemness properties, suggesting that Tie-1 may have potential clinical applications in targeting cancer stem cells.

In the current study, we also showed that sTie-1 effectively distinguished CSCC from healthy controls and patients with CIN, and was especially efficacious in distinguishing cervical cancer patients from healthy controls. The role of sTie-1 concentrations in differentiating cervical cancer from CIN was likewise statistically significant. The AUC value of the ROC curve for sTie-1 (detecting cervical cancer as compared with healthy controls) was 0.846, which was higher than that of sAng-2 (0.769) and the sAng-1:sAng-2 ratio (0.719), as reported in our previous study [16]; the corresponding sensitivity and specificity values were 78.7 and 94.5%, respectively, indicating that, compared with sAng-2 and the sAng-1/sAng-2 ratio, sTie-1 levels may be a more sensitive and specific novel diagnostic measure for distinguishing healthy controls, CIN cases, and cervical cancer cases, with the strongest results observed for distinguishing cervical cancer patients from healthy controls. In metastatic breast cancer patients, plasma Tie-1 levels were statistically significantly decreased as compared with pre-operation after 6 weeks of chemotherapy [10], and a recent *in vitro* study indicated that the overexpression of Tie-1 in multiple ovarian cancer cell lines decreased cisplatin sensitivity indicating that this may be a key gene inducing platinum resistance [17]. Thus, we conclude that sTie-1 may be a potential novel serum marker for monitoring tumor progression in cervical cancer patients, similar to CA125 in ovarian cancer, and may be a marker for platinum resistance. These questions merit additional thorough investigation in future research.

In addition to the substantial strengths of our study, we acknowledge some limitations. First, the sample size in the current study was insufficient, especially for examining associations with cervical adenocarcinoma; this may have led to some selection bias and reductions in statistical power. Second, the follow-up time for our patients with cervical cancer was relatively short. In the future, expanding the sample size of enrolled patients and healthy controls and extending the follow-up time could effectively validate our results and provide more conclusive, rigorous, and generalizable findings.

## Conclusion

Our findings revealed that, as the severity of cervical lesions increased, the level of serum Tie-1 gradually increased, and that sTie-1 levels can effectively distinguish cervical cancer patients from healthy controls. Thus, sTie-1 levels may be associated with the growth and progression of cervical cancer. We also found that high sTie-1 levels predict a poorer prognosis in cervical cancer patients. Therefore, sTie-1 may be a valuable biological marker for predicting the progression and prognosis of cervical cancer.

## Data Availability

The original contributions presented in the study are included in the article/Supplementary Material, further inquiries can be directed to the corresponding author.
